# The Role of Connexin 43 in Lung Disease

**DOI:** 10.3390/life10120363

**Published:** 2020-12-19

**Authors:** Julie A. Swartzendruber, Bruce J. Nicholson, Ashlesh K. Murthy

**Affiliations:** 1 Department of Microbiology and Immunology, College of Graduate Studies, Midwestern University, Downers Grove, IL 60515, USA; 2 College of Veterinary Medicine, Midwestern University, Glendale, AZ 85308, USA; amurth@midwestern.edu; 3 Department of Biochemistry, School of Medicine, University of Texas Health Science Center, San Antonio, TX 78299, USA; NicholsonB@uthscsa.edu

**Keywords:** asthma, gap junction proteins, connexin 43, acute respiratory distress syndrome, mitochondria

## Abstract

The term lung disease describes a broad category of disorders that impair lung function. More than 35 million Americans have a preventable chronic lung disease with high mortality rates due to limited treatment efficacy. The recent increase in patients with lung disease highlights the need to increase our understanding of mechanisms driving lung inflammation. Connexins, gap junction proteins, and more specifically connexin 43 (Cx43), are abundantly expressed in the lung and are known to play a role in lung diseases. This review focuses on the role of Cx43 in pathology associated with acute respiratory distress syndrome (ARDS), chronic obstructive pulmonary disease (COPD) and asthma. Additionally, we discuss the role of Cx43 in preventing disease through the transfer of mitochondria between cells. We aim to highlight the need to better understand what cell types are expressing Cx43 and how this expression influences lung disease.

## 1. Introduction

**Figure 1:**
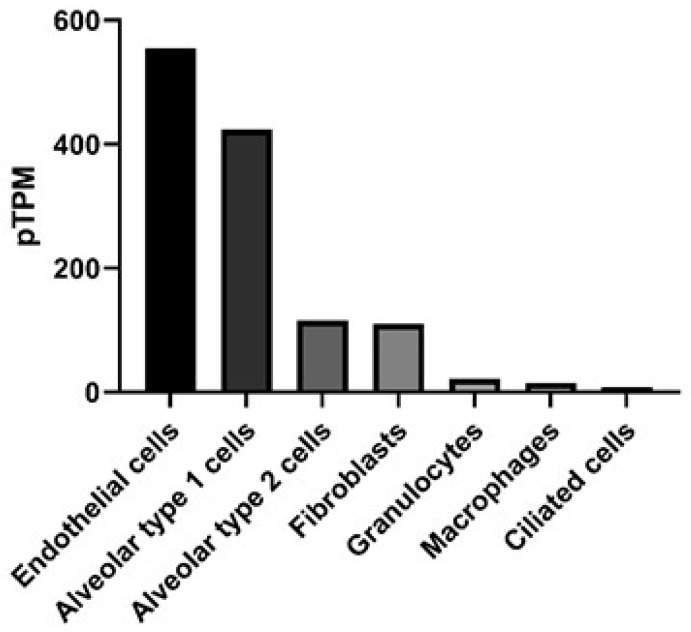
Gene expression in the lung for gap junction protein, alpha (*GJA1*) that encodes for the protein connexin 43. Data presented as protein-transcripts per million (pTPM) with *GJA1* expression in RNA-seq data obtained from The Human Protein Atlas available from http://www.proteinatlas.org [27].

Connexins (Cx) are gap junction proteins involved in cell-to-cell communication. Connexins assemble in the plasma membrane to form gap junctions by assembling six connexin subunits to form a connexon [1,2]. Connexons bridge two neighboring cells by creating a channel between the two cells that mediate the transport of ions, nutrients, signaling molecules, as well as miRNAs between the two cells [3,4,5,6]. There are currently at least 20 mouse and 21 human connexin genes. The connexins they encode can assemble to form homomeric or heteromeric connexons [7]. Homomeric connexons utilize a single connexin protein, while heteromeric connexons involve the assembly of two or more connexin protein subunits to form a connexon [8], but the rules regarding connexin compatibility are still unclear. More progress has been made in assessing which connexins can interact heterotypically (i.e., between connexons expressed in different cells) [9]. This is of great significance in terms of which regions of a tissue communicate, for instance, Cx43 forms heterotypic-connexons with some but not all connexins. In a normal lung Cx32 is expressed by Type II alveolar cells and does not form heterotypic-connexons with Cx43. However, in response to lung injury Cx46 expression is increased on Type II alveolar cells, which can form heterotypic-connexons with Cx43, thereby increasing the potential for communication between the alveolar epithelium [10,11]. The subunit composition of a gap junction channel is known to influence its channel characteristics. For example, connexin composition will influence the size or charge of molecules that can be transported through the gap junction [12,13,14]. Differences in the charge-selectivity of different connexin channels has even been shown to lead to electrical rectification (i.e., asymmetrical current flow between cells) of some heterotypic channels, like those between Cx26 and 32 [15]. Differential permeabilities have been demonstrated for various metabolites, including adenosine triphosphate (ATP), which was found to more easily pass through connexons formed by Cx43, when compared to connexons consisting of Cx32 [16,17]. Additional forms of connexin regulation include post-translation modifications such as phosphorylation, and at the transcriptional level, as connexins have very rapid turnover, with a half-life of 1–5 h [18]. Ubiquitylation of connexins is responsible for targeting the connexin for endosomal degradation, while phosphorylation can support localization, degradation and channel size [19,20,21]. *Pseudomonas aeruginosa,* for example can trigger cells through toll-like receptor 2 (TLR2) to close Cx43 containing gap junctions, through tyrosine phosphorylation that closes the channel, probably by C-terminal occlusion of the pore [22] and prevents further exchange of inflammatory signals [23]. We are interested in understanding the development and pathogenesis of inflammation in the lung, specifically the role of Cx43. Connexin expression is known to vary by cell type in the lung [24] and has been reported in all murine respiratory tissues [25]. Human lungs exhibit detectable Cx43 across multiple cell types, including epithelial and endothelial cells, determined by RNA-seq, as shown in Figure 1 [26].

## 2. Connexin 43 in Lung Development

Cx43 is known to be important for the development of organs such as the brain, heart, kidney and lung, as evidenced by neonatal death in mice with homozygous deletion of Cx43, due to occlusion of the pulmonary circulation due to malformation of the heart [28]. Cx43 is expressed in the mouse embryo as early as day 14.5 and in the adult airway epithelium, Type I and Type II alveolar cells, pulmonary endothelium, lung fibroblasts and lung smooth muscle cells [24,29]. Cx43 deficient mice have narrowed and delayed development of lung alveoli in neonate mice due to reduced expression of important factors such as surfactant protein C and alpha-smooth muscle actin [30]. Gap-junctions are critical for the intercellular propagation of calcium signals, which helps regulate many lung functions, such as ciliary movement and surfactant secretion [24]. In the absence of Cx43 expression, calcium transfer between alveoli and lung capillaries is prevented [31]. Cx43 may play a role in lung repair, as suggested by increased Cx43 expression by alveolar epithelial cells following radiation-induced pulmonary fibrosis [32], as well as increased Cx43 expression by pulmonary endothelial cells following repeated thrombin treatment that mimics chronic inflammation [33]. The understanding of Cx43 in development and repair processes in the lung is of interest to better diagnose and treat disorders of respiratory failure.

## 3. Involvement of Connexin 43 in Respiratory Failure

Respiratory failure is a broad class of conditions that results in impaired gas exchange. Previous evidence has established the importance of Cx43 in the development of the lung and specifically the lung alveoli, therefore, it is reasonable to predict that Cx43 plays a role in a variety of respiratory failure conditions. For this review we are going to focus on the role of Cx43 in acute respiratory distress syndrome (ARDS), chronic obstructive pulmonary disease (COPD) and asthma, summarized in Table 1. Roles for Cx43 in other forms of respiratory failure are discussed elsewhere [24,34,35,36,37].

## 4. Connexin 43 in Acute Respiratory Distress Syndrome

ARDS is a broad category of diseases with significant morbidity and mortality. Mortality rates for ARDS are approximately 40% with limited effective treatment options. The definition for ARDS has fluctuated over the last several decades and was previously a subset of Acute Lung Injury (ALI). ALI and ARDS are frequently the result of bacterial pneumonia or sepsis. ALI was removed in 2012 as a categorization of respiratory failure, but is still used in historical literature and to describe the disease models of ARDS or ALI in mice. Current definition of ARDS is the Berlin Definition and is based on the degree of hypoxemia and respiratory function [43]. Lung function is significantly impaired in ARDS because of damage to the alveolar epithelium and capillary endothelium. This damage results in impaired gas exchange, pulmonary hypertension and decreased lung compliance [44]. Treatment for ARDS is mechanical ventilation, which can lead to further complications including ventilator-induced lung injury [45]. The respiratory failure found in ARDS can be attributed to impaired oxygen exchange caused by damage to the alveolar-capillary barrier, as well as increased permeability of the airway endothelial and epithelial cells, leading to infiltration of neutrophils [46]. Gap junction proteins, specifically Cx43, are important in maintaining the endothelial and epithelial cell barriers. The mechanism for this is still unclear, but appears likely to involve a role in the localization of tight junction and possibly adhesive proteins to sites of cell interfaces [47,48]. However, the role of gap junction proteins is more complex given that signaling between invading immune cells and the endothelium, mediated by either heterotypic gap junctions, [49,50] or release of ATP through hemichannels [51], has been shown to significantly modulate the barrier function of vascular endothelia, discussed in relation to neutrophil invasion below. Therefore, understanding connexin expression levels and function in models of ALI, may inform future treatment options for ARDS. We know from in vitro studies that Cx43 expression is essential for communication between alveolar epithelial cells [52]. Common mouse models of ALI involve the intranasal administration of bacteria or bacterial product, such as lipopolysaccharide (LPS), or acid aspiration [53]. Sarieddine et al. found that mice with reduced Cx43 (+/−) have decreased neutrophils in the bronchial alveolar lavage fluid (BALF) following an intratracheal challenge with *Pseudomonas aeruginosa* LPS compared to wild-type Cx43 (+/+) mice [38]. This suggests a role for Cx43 in worsening the symptoms of ALI/ARDS. This is supported by the independent observation that mice with constitutively open Cx43 gap junction channels (Cx43K258stop mice with a truncated form of Cx43) have increased neutrophils, while blocking Cx43 with mimetic peptides (43Gap26 and 43Gap27) results in reduced neutrophil recruitment to the lungs of rats [38]. Cx43 is ubiquitously expressed in the lung, so loss of Cx43 function could be attributed to reduced expression in either the airway epithelium or alveolar cells. However, Cx43 is also expressed by neutrophils, and heterotypic gap junctional coupling between invading cells and the endothelium has been shown to promote invasion [50]. Interestingly, most of the focus in the literature on Cx43 in neutrophils has been on its role as a hemichannel that exists in the membrane prior to forming gap junction channels with another cell. Eltzschig et al. demonstrated that dephosphorylation of Cx43 in neutrophils allows for opening of these Cx43 hemichannels, and the release of ATP [51], which is highly inflammatory [54], and has been seen to promote barrier function in endothelial cells [51], in contrast to the disruption of the barrier function associated with heterotypic gap junctions. This data all supports the hypothesis that Cx43 represents a potential therapeutic target for restoring respiratory function, possibly by preventing the influx of neutrophils. It also highlights that when Cx43 levels are linked to a specific phenotype, it is important to establish if it is due to gap junction coupling between cells, or release of metabolites (such as pro-inflammatory ATP) to the media through hemichannels, a distinction that is only recently being made in the literature.

To better understand the kinetics of Cx43 expression, Kandasamy et al. found that Cx43 expression was maintained in the lungs of rats following challenge with LPS for the first 4 h, but then dropped progressively between days 1 and 5 (to only 25% of control levels), before returning to normal by day 14 [55]. This expression pattern was found in both venules and capillaries and suggests that Cx43 has distinct roles in steady state compared to acute phases of inflammation, but may inhibit the resolution phase from days 5–14. These changes in Cx43 were accompanied by inverse changes in expression of a critical lung adhesion molecule, VE-cadherin, which was also found to increase in response to knockdown of Cx43, by shRNA [55]. While expression levels are informative, it is important to remember that Cx43 surface expression is short-lived and the protein is internalized within 1–5 h [56], and that gap junction coupling of cells has been observed to be regulated by internalization of the protein during cancer progression [57]. Better readouts for Cx43 function involve measuring the extent of molecule transfer between cells or disease outcomes. For example, when Cx43 expression is reduced in vascular endothelial cells there is reduced vascular barrier permeability, while increased Cx43 expression results in increased permeability [33,55,58]. In addition to impacting vascular permeability, Cx43 spreads proinflammatory signals by shuttling Ca^2+^ between cells, possibly indirectly through its hemichannel function [5]. Inhibitors of Cx43 or Cx43 deficient endothelial cells exhibit reduced inflammation in ALI, due to reduced Ca^2+^ spread, and Ca^2+^ induced expression of P-selectin [31]. Reduced P-selectin expression would reduce extravasation and contribute to the reduced neutrophilia found in Cx43 (+/−) mice in models of ALI, as described above.

In addition to impacting the vascular endothelium, Cx43 expression is important for the function of alveolar macrophages and epithelial cells, as demonstrated by knocking out Cx43 in both cell types resulting in increased disease severity [39]. Specifically, Cx43 was essential for suppressing excess inflammation through Ca^2+^-dependent signaling between alveolar macrophages and the alveolar epithelium [39]. Cx43 expression is also known to be important for macrophage phagocytosis of bacteria [59]. Additionally, Cx43 expression by lung fibroblasts has been shown to be involved in LPS-induced apoptosis [60]. All of this supports the importance of Cx43 in lung function homeostasis, signaling the alarm during acute injury through alveolar macrophages and epithelial cells to suppress inflammation and increasing inflammation through expression by vascular endothelial cells. Insights into the specific roles of Cx43 in ALI/ARDS, particularly how Cx43 expression by various cell types influences disease, is necessary to unravel the complexities of these prior observations and provide critical information that will inform development of novel treatments for patients who suffer complications from prolonged inflammation, or delayed resolution. In particular, it is clear that targeting Cx43 would have to be done in a nuanced manner, given that, depending on cell type responsible for disease pathology, it can play roles in both promoting and suppressing aspects of inflammatory lung disease.

## 5. Connexin 43 in Chronic Obstructive Pulmonary Disorder

COPD is characterized by persistent respiratory symptoms as a result of inhalation of particles that contributes to inflammation and injury of epithelial cells. The most common risk factor associated with COPD is exposure to cigarette smoke, making COPD a preventable disease [61]. Chronic inflammation due to COPD is associated with significant reductions in quality of life, as well as substantial health care use [62,63]. Symptoms of COPD include difficulty breathing, wheezing and persistent cough, the results of thickening of the airway smooth muscle and connective tissue that restricts airflow. Damage to alveolar epithelial cells impairs gas exchange and over time results in cyanosis of mucosal membranes. Treatments for COPD, such as bronchodilators and corticosteroids, reduce symptoms, but have been unable to reverse damage to lung function. Modeling of COPD in mice is achieved through exposing mice to cigarette smoke. Nicotine is a primary component of cigarettes that has addictive properties, as well as direct effects on the respiratory system, such as altering respiration and reducing elastin in the lung [64]. Cx43 expression is downregulated in response to nicotine exposure in human endothelial cells, but unaltered in human epithelial cells [40,41,42]. The major intercellular junctions that are disrupted in COPD are tight junctions, but given their close association with gap junctions, and sharing of several accessory proteins like zona occludens-1 (ZO-1), an analysis of disruptions of gap junctions is certainly warranted [65]. The endothelium is crucial for providing barrier protection in the lung and reduced Cx43 is likely part of a larger signaling cascade that results in the breakdown of alveolar barrier function.

## 6. Connexin 43 in Asthma

Asthma is the result of an inappropriate response to innocuous substances that triggers airway obstruction, shortness of breath and labored breathing. The incidence of asthma has been on the rise and affects approximately 8% of the United States population [66]. Risk factors for developing asthma include genetic factors and environmental exposure [67]. Mechanistically, asthma can be classified into occupational asthma, allergic, and non-allergic asthma. Occupational asthma is the result of exposure in the working environment to harmful irritants, such as chemicals or dust, that trigger constriction of the airway. Allergic asthma involves sensitization to specific antigens, referred to as allergens. Common allergens include pollen, pet dander and dust mites. Exposure to allergens results in the production of allergen-specific antibodies, specifically Immunoglobulin E (IgE). IgE binding to the allergen initiates an inflammatory cascade that induces inflammation characterized by mast cell degranulation and an influx of eosinophils and cytokine release. Non-allergic asthma includes individuals who have difficulty breathing following exercise, stress or following viral respiratory infections. The impaired air flow in asthmatics is the result of thickening of airways, increased mucus and increased smooth muscle cell mass. These features all contribute to the characteristic labored breathing and shortness of breath [68]. Treatment for asthma can vary depending on what triggers the airway obstruction. Patients with asthma triggered by environmental exposures are often advised to avoid the offending allergen. Other treatment options include inhalers that contain corticosteroids, beta2-agonists and anti-leukotrienes that target the inflammatory signals released from mast cells and eosinophils. Allergy shots are a type of immunotherapy that seeks to reduce the symptoms triggered by allergens by creating tolerance of the allergen [69]. Allergy shots and other forms of immunotherapy can be time consuming and expensive [70].

Research into the role of Cx43 in asthma has been primarily focused on understanding its role in allergic asthma. Cx43 is known to be upregulated in a model of ovalbumin-induced allergic lung inflammation, occurring broadly within 1 h following OVA challenge [71]. In addition to Cx43 levels correlating with increased inflammation, Cx43 inhibition with the blocking peptide Gap26 reduced airway hyper responsiveness [72]. Blocking Cx43 function also reduced other hallmark features of allergic asthma, including eosinophil infiltration, Th2 cytokine levels in the bronchial alveolar lavage fluid and serum OVA-specific IgE levels. However, in this study by Huang et al., the cell type influenced by blocking Cx43 is unclear. Cx43 is ubiquitously expressed by resident cells of the lung, such as airway epithelium, alveolar cells and lung fibroblasts (Figure 1). In asthmatics there is a failure to properly repair epithelial cell damage, and excessive growth factor production by fibroblasts [73]. Cx43 is also expressed by cells critical for the allergic process, such as mast cells, which release numerous mediators that contribute to bronchoconstriction, vascular leakage and recruitment of inflammatory cells [74]. It is unclear from the present studies if the reduced allergic disease in the absence of Cx43 function is due to local cell expression of Cx43, or impaired Cx43 on recruited cells, such as mast cells. With regard to inflammatory processes, there is growing emphasis on the role of Cx43 hemichannels, which, as noted above, release ATP, which can be highly inflammatory. This is consistent with the effects of Gap26, which primarily blocks hemichannels, although prolonged application also reduces gap junction functions [75]. More definitive blockers of hemichannel and gap junction functions in the form of dominant negative Cx43 constructs are now available, and would help to definitively show which functions of Cx43 contribute to these inflammatory events. Similarly, it is unclear in the pathogenesis of asthma if resident airway cells or recruited inflammatory cells are the primary drivers of disease [76]. Regardless, Cx43 is potentially a relevant player in both categories of cells.

## 7. Connexin 43-Induced Transfer of Mitochondria

Cx43 is known to be important for ARDS, COPD and asthma, as already described, and the mechanism appears to at least be in part due to propagation of calcium, and possibly other signals, between cells via gap junctions, and ATP release through hemichannel opening, as the primary function of Cx43 in lung disease. It should also be noted that, in addition to small molecules, gap junctions can also mediate the transfer of antigenic peptides between cells (potentially spreading inflammatory responses to infection [77] and miRNAs [78], that can also regulate many processes. Beyond that, a recent work shows Cx43 also induces formation of nanotubes, by an undefined mechanism, and that these nanotubes can transfer mitochondria between cells [79]. The transfer of mitochondria from stem cells is a promising therapeutic that has demonstrated potential in lung disease, as well as cancers. Mitochondria were shown to be transferred from bone marrow-derived stromal cells to the alveolar epithelium in mice [80], in a Cx43-dependent manner, thus providing protection against LPS-induced ALI. Similarly, Yao et al. found that mitochondria from human induced pluripotent stem cells were transferred to lung epithelial cells, reducing OVA-induced asthma inflammation, a process that was also dependent on Cx43 expression [79]. Mitochondria play a critical role in disease processes due to its generation of ATP and its management of reactive oxygen species. During asthma, there is an increase in mitochondrial dysfunction and the hallmark asthma cytokine, IL-4, is associated with increased mitochondrial damage [81]. Impaired mitochondrial dysfunction has also been observed in COPD and the transfer of human induced pluripotent stem cell-derived mesenchymal stem cells (iPSC-MSCs) into a rat challenged with cigarette smoke exhibited reduced alveolar destruction, lung fibrosis, as well as transfer of mitochondria to airway epithelial cells [82]. Many of the symptoms of lung disease are the result of damage to the function and health of endothelial and epithelial cells. Increasing Cx43 expression in order to promote the transfer of energy producing mitochondria to damaged cells could provide a novel approach to improving symptoms of lung disease.

## 8. Discussion

Our understanding of the role of Cx43 in lung disease requires further investigation. It is clear that cell types respond differently to Cx43 expression, and it is also clear that Cx43 can influence processes through the direct intercellular channels of gap junctions, as well as by releasing signals like ATP to the extracellular milieu via hemichannels. Future work will need to investigate the effects of Cx43 expression in specific cell types and how this influences disease outcomes. It will also be important to use more refined tools that can distinguish the role of hemichannels and gap junctions, by using specific mutants that only affect one of these channel isoforms. Finally, it’s important to consider, in all of the above referenced studies, that looking at just one connexin, for example Cx43, narrows the window of reality. For example, it has been shown that some of the defects in lung development seen in Cx43 KO mice, can be restored with the induced expression of Cx32 or Cx40 [83]. Cx43 has been proposed as a therapeutic target in a range of diseases, including cancer, skin diseases, corneal wounds and cardiac ischemic injury. However, while blocking the function of Cx43 may be beneficial in preventing growth of tumor cells or in improving wound-healing, it is going to be important to understand the side-effects that may arise by blocking Cx43 function, both due to its multiple effects within the target tissue, but also effects on other systems. Potentially more promising, is the use of connexin expression as a biomarker for disease. Evidence from the cancer field has identified Cx43 expression as a prognostic indicator [84] and treatment with connexin-mimetic peptides can suppress inflammation [38]. Treatment of lung disease is challenging, but increasing our understanding of the cellular mechanisms surrounding gap junctions, specifically Cx43, will provide the knowledge necessary for informing which stages of the disease might be affected most by interrupting or promoting Cx43 activity, and which of its functions are critical. Like any therapeutic or diagnostic target, it is most effective when the nuances of its roles are first defined.

## Figures and Tables

**Table 1:** Connexin 43 expression in lung disease.

Cell Type	Cx43 Expression	Outcome	Reference
Acute Lung Injury Models			
Whole Body	KO	Lethal	[28]
Whole Body	Het	Reduced Disease	[38]
Whole Body	Increased	Increased Disease	[38]
Alveolar Macrophages	KO	Increased Disease	[39]
Alveolar Epithelium	KO	Increased Disease	[39]
Vascular Endothelium	KO	Reduced Disease	[31]
COPD Models			
Endothelial	Normal	Cx43 reduced	[40,41]
Epithelial	Normal	Unchanged	[42]
OVA-Asthma Models			
Whole Body	Normal	Cx43 elevated	[38]
Whole Body	Reduced	Reduced Disease	[38]

Knockout (KO), Heterozygous (Het), Ovalbumin (OVA).
